# The Dual Role of Macrophage Extracellular Traps in Host Defense and Disease: Mechanisms and Therapeutic Implications

**DOI:** 10.3390/biom15091220

**Published:** 2025-08-24

**Authors:** Zhiyu Chen, Fei Gao

**Affiliations:** Department of Emergency, The Affiliated Wuxi People’s Hospital of Nanjing Medical University, Wuxi People’s Hospital, Wuxi Medical Center, Nanjing Medical University, Wuxi 214023, China; chenzhiyu1224@stu.njmu.edu.cn

**Keywords:** macrophage, infection, immune, therapy

## Abstract

Macrophage extracellular traps (METs), which are extracellular immune structures released by macrophages, consist primarily of double-stranded DNA, histones, elastase, matrix metalloproteinase, myeloperoxidase, and other components. Recent studies have found that various pathogens (such as bacteria, fungi, and parasites) and environmental pollutants could induce the formation of METs through different mechanisms to help the host resist infection. However, accumulating evidence suggests that METs play a double-edged role in immune response, enhancing host defense while potentially contributing to tissue damage under certain pathological conditions. This review summarizes the mechanisms underlying the formation of METs, including different pathways triggered by various pathogens and environmental pollutants. We also discussed the role of METs in respiratory diseases, autoimmune diseases, metabolic diseases, tumors, and transplantation and injury, as well as recent advances in MET-targeted drugs, aiming to provide new insights for improving treatment strategies of relevant diseases.

## 1. Introduction

Macrophage extracellular traps (METs) were first reported in 2010 [1]. Similar to neutrophil extracellular traps (NETs) [2], METs play a key role in pathogen capture and clearance, as well as in various immune responses. Recent studies have revealed that macrophages could form METs in response to various pathogenic stimuli, contributing to a hosts defense against infection [3,4]. However, METs exhibit an inherently double-edged role in immune responses, facilitating host defense against pathogens while potentially aggravating tissue damage under certain pathological conditions [3,5]. This review summarizes the mechanisms underlying MET formation, the recent advances in understanding the roles of METs in various diseases, and the development of drugs targeting METs.

## 2. Structure and Formation Mechanisms of METs

NETs are fibrous structures released from activated neutrophils, primarily composed of DNA, histones, and antibacterial proteins like neutrophil elastase and myeloperoxidase [2]. In comparison, METs are web-like structures released by activated macrophages. The core component of METs is a deoxyribonucleic acid (DNA) scaffold decorated with histones and various antimicrobial proteins, such as myeloperoxidase [6], elastase [4], and lysozyme [6]. The DNA in METs originates from both nuclear and mitochondrial sources. Histones can undergo citrullination mediated by peptidyl arginine deiminases 2 and 4 (PAD2 and PAD4), promoting chromatin decondensation through the alteration of histone charge [7]. In addition, METs are enriched with matrix metalloproteinases (MMPs) [8], which are closely related to antimicrobial processes [9]. Similarly, lysozyme and lactoferrin play important roles in microbial killing. Several studies have shown that the mechanisms by which various pathogens trigger and promote the formation of METs differ [10,11]. For example, METs induced by *Mycobacterium tuberculosis* are dependent on the 6 kDa early secretory antigen target (ESAT-6), a virulence factor specific to the *M. tuberculosis* complex and commonly used as a detection antigen to identify *M. tuberculosis* infections [10]. However, METs triggered by *Porphyromonas gingivalis* (*P*. *gingivalis*) involve mitochondrial DNA release mediated by calcium influx [11].

To date, METs have been categorized into two primary types, one type mediated by the production of reactive oxygen species (ROS) dependent on NADPH oxidase, and the other mediated by ROS-independent mechanisms. The ROS-dependent pathway is commonly observed in infections caused by Gram-positive bacteria such as *Staphylococcus aureus* (*S. aureus*) and *Streptococcus agalactiae* (*S*. *agalactiae*) [3,12]. In this process, pathogens activate NADPH oxidase through Toll-like receptors, resulting in excessive ROS production that promotes chromatin decondensation and nuclear envelope rupture, ultimately leading to MET release via plasma membrane permeabilization [3,12]. The ROS-independent pathway is more frequently observed in infections caused by Gram-negative bacteria, such as *Escherichia coli* (*E. coli*) and mycobacteria. The underlying mechanisms involve calcium signaling pathways, the AMPK/ULK1/mTOR autophagy-related pathway, and histone modifications mediated by PADs [11,13,14]. For example, MET formation induced by *Salmonella* is promoted through activation of the AMPK signaling pathway by the bioactive compound biochanin A (BCA), whereas *P*. *gingivalis* induces METs through direct DNA extrusion driven by an increase in intracellular calcium levels [11,13].

Meanwhile, several studies have shown that the polarization state of macrophages significantly influences MET release. M1 macrophages, characterized by their pro-inflammatory phenotype, are known to secrete pro-inflammatory cytokines, including interleukin (IL)-1, IL-6, IL-12, IL-23, and tumor necrosis factor-alpha (TNF-α) [15]. These cytokines, in conjunction with pro-inflammatory stimuli such as interferon-gamma (IFN-γ), lipopolysaccharide (LPS), and TNF-α present within the M1 macrophage microenvironment, are capable of activating signaling pathways implicated in the formation of METs [16]. Specifically, stimuli such as IFN-γ and LPS are instrumental in the activation of M1 macrophages, and these activation signals are intricately associated with the induction of MET formation. The process of MET formation is typically linked to the presence of ROS and the influx of calcium ions. Upon activation, M1 macrophages exhibit an increased propensity to generate ROS, and their intracellular calcium signaling pathways are more readily activated, which further contributes to MET formation [16]. In contrast, M2-polarized (anti-inflammatory) macrophages could significantly reduce MET release [17]. Microenvironmental stimuli (such as statins that inhibit cholesterol biosynthesis) and pathogen-derived toxins (such as hemolysins) can regulate MET generation by activating downstream signaling pathways via specific receptors, like CD18 [1,18]. Unlike many other immunoregulatory factors, METs play a dual role in immune responses across various diseases. During macrophage extracellular trapsosis (METosis), a form of programmed cell death associated with MET formation, the coordinated process of chromatin decondensation and granule content release facilitates pathogen entrapment, but may also exacerbate tissue damage when excessively activated, such as acute kidney injury (AKI) and chronic pulmonary diseases [19,20,21].

## 3. Mechanistic Studies on MET Formation Induced by Various Stimuli

### 3.1. Mechanisms Underlying MET Formation Induced by Pathogenic Microorganisms

Pathogenic microorganisms can induce the release of METs through multiple mechanisms, which play a critical role in enhancing host immune defense. The formation of METs is a common phenomenon in bacterial infections. In 2021, Monaco et al. reported that *Salmonella typhimurium* (*S*. *typhimurium*) could induce MET release in the murine macrophage cell line J774A.1, facilitating the clearance of pathogens [22]. Qian et al. further demonstrated that *Salmonella* promoted the formation of METs by inducing the release of 5’-nucleotidase [23]. METs enhanced the host immune response against *Salmonella*, effectively restricting bacterial dissemination and establishing a robust immunological barrier within host cells [23]. A recent study found that *Mycoplasma bovis* could activate METs through a mechanism dependent on the production of NADPH oxidase and ROS [24]. Baz et al. observed the formation of extracellular web-like structures by co-culturing *Mycoplasma bovis* with bovine macrophages for six hours, as visualized by dual staining with SYTOX Green and Hoechst 33342. Co-localization of structural and marker proteins validated the identity of the observed extracellular reticular network as METs [24]. Furthermore, the study demonstrated that MET formation contributed to the host-mediated clearance of *Mycoplasma bovis*, indicating that METs played an active role in the immune response [24].

These findings suggest that METs contribute to host defense against bacterial infections, while their roles in infections caused by other pathogens involve more complex mechanisms. *Candida albicans* (*C. albicans*) can induce MET formation by activating macrophage immune responses [6,25]. In 2014, Liu et al. identified that *E. coli* and *C. albicans* stimulated the release of METs [6]; however, the microbicidal activity of METs induced by *C. albicans* appears to be relatively limited, with the primary function focused on restricting the dissemination of invading microorganisms at the location of infection rather than directly exerting potent pathogen-killing effects. In 2019, Loureiro et al. reported that *C. albicans* employed an immune evasion strategy by secreting DNases to degrade METs and compromise host immune surveillance [26]. In 2022, Olivier et al. performed infection assays utilizing murine bone marrow-derived macrophages (BMDMs) and a transgenic *C. albicans* strain expressing the fluorescent protein dTomato [25]. The study revealed that *C. albicans* escape was associated with the host cell membrane damage and the activation of two cell death pathways caused by candidalysin—Gasdermin D-mediated pyroptosis triggered by the pore-forming toxin and the formation of METs [25]. However, although this study demonstrated that host cell death was associated with *C. albicans* escape, inhibiting cell death did not significantly reduce escape efficiency. The findings suggest that *C. albicans* might utilize additional mechanisms to evade host immune responses.

Similarly, *Mycobacterium tuberculosis* (*M. tuberculosis*) can also promote immune evasion by inducing the release of METs [27]. Wong et al. isolated primary human macrophages from the peripheral blood of healthy donors with the *M. tuberculosis* infection [27]. They observed that IFN-γ enhanced the formation of METs induced by *M. tuberculosis* and further demonstrated that this process was dependent on the ESX-1 secretion system, a distinctive type VII secretion system present in *M. tuberculosis*. As a critical virulence factor, this system is able to impair lysosomal function, recruit immune components, and suppress host immune responses [28,29]. Moreover, they found that IFN-γ further enhanced ESX-1-mediated macrophage necrosis, which played a critical role in facilitating the long-term survival and dissemination of *M. tuberculosis* within the host [27]. The study also revealed that *M. tuberculosis* could alter macrophage polarization to manipulate the immune response in support of survival, leading to immune dysregulation and the establishment of persistent chronic infection. Moreover, Kalsum et al. found that infection with the corded phenotype of *M. tuberculosis* induced the formation of METs in host cells through the action of ESAT-6 [10]. In contrast to Mycobacterium species, infection with *S. aureus* triggered the release of METs with stronger bactericidal activity, with more effective capture and elimination of invading pathogens [3].

Nontypeable *Haemophilus influenzae* (NTHi) can induce macrophage extracellular trap-like structures through a ROS-dependent mechanism, concurrently promoting protease co-expression. Dousha et al. confirmed that NTHi-stimulated alveolar macrophages released extracellular chromatin-DNA networks co-expressing MMP-12—a protease critically involved in emphysema pathogenesis [30]. MET formation directly correlates with significantly elevated ROS levels, a process effectively suppressed by the ROS inhibitor apocynin [31]. DNase treatment degrades extracellular DNA, reducing NTHi-induced METs by over 90% [31]. These findings demonstrate that NTHi activates a ROS-METs-MMP12 pathway in macrophages, driving pulmonary oxidative stress and protease imbalance, while DNase disrupts this pathological cascade [30,31].

METs are also implicated in the immune response against parasitic infections. Liao et al. treated Sema4D-knockout murine macrophages using schistosome egg-derived extracellular vesicles (E-EVs) highly expressing *S*. *japonicum* microRNA-71a (Sja-miR-71a, a microRNA secreted by *S. japonicum*) [32]. Sema4D, also known as CD100, functions as a membrane-anchored protein but can be also released as a soluble extracellular fragment through proteolytic cleavage [33]. During normal immune responses, Sema4D activates proinflammatory cytokines (such as TNF-α and IL-6) to enhance bactericidal activity [34]. A previous study demonstrated that Sja-miR-71a, suppressed the formation of METs and NETs by downregulating Sema4D and consequently upregulating IL-10 [32]. In Sema4D-deficient macrophages infected with *S. japonicum*, Peroxisome Proliferator-Activated Receptor Gamma (PPAR-γ) and IL-10 expression were significantly increased, accompanied by a marked reduction in MET and NET formation [32]. Upon further treatment with an IL-10 neutralizing antibody, the inhibitory effect of Sja-miR-71a on Phorbol-12-Myristate-13-Acetate (PMA)-mediated MET and NET formation was notably attenuated [32]. These findings suggest that Sja-miR-71a carried by *S. japonicum* egg-derived EVs suppresses MET formation through the Sema4D/PPAR-γ/IL-10 axis, thereby contributing to immune evasion.

Some probiotics, such as Bacillus subtilis and Bacillus licheniformis, can also promote the release of METs by activating macrophages [35]. In murine models, these probiotics enhanced the host’s ability to resist S. aureus infections by increasing MET formation. P. gingivalis has also been shown to induce the formation of METs [11]. Macrophages pretreated with P. gingivalis exhibited a significantly increased release of METs. This process appeared to be associated with increased intracellular Ca^2+^ levels, but is independent of ROS production and Rapidly Accelerated Fibrosarcoma (RAF)/Mitogen-Activated Protein Kinase Kinase (MEK)/Extracellular Signal-Regulated Kinase (ERK) pathway phosphorylation [11].

### 3.2. Mechanisms of Environmental Pollutant-Induced MET Formation

Recently, researchers in the field of environmental engineering have extensively investigated how key components of environmental pollutants affect macrophage function, revealing multiple mechanisms by which these pollutants modulate the formation of METs to disrupt host immune homeostasis [36,37,38,39]. Cui et al. reported that environmental pollutants such as black carbon (BC) and its oxidized derivatives, 1,4-naphthoquinone-coated black carbon (1,4 NQ-BC), ozone-oxidized black carbon particles, and various types of nanoparticles could significantly regulate MET formation in macrophages [37,38]. These pollutants interfered with macrophage immune function through multiple pathways, notably by suppressing MET formation via the necroptosis pathway. For instance, 1,4 NQ-BC induced necroptotic cell death in macrophages by increasing intracellular levels of ROS and calcium, thereby significantly inhibiting the release of METs. This mechanism not only impaired the basic immunological functions of macrophages but also compromised their ability to capture and eliminate pathogens, ultimately weakening the host immune response [37]. Similarly, BC promoted necroptosis by enhancing ROS accumulation, thus affecting MET formation. Additional studies have confirmed that the inhibition of necroptosis could restore the ability of macrophages to form METs [39].

**Figure 1:**
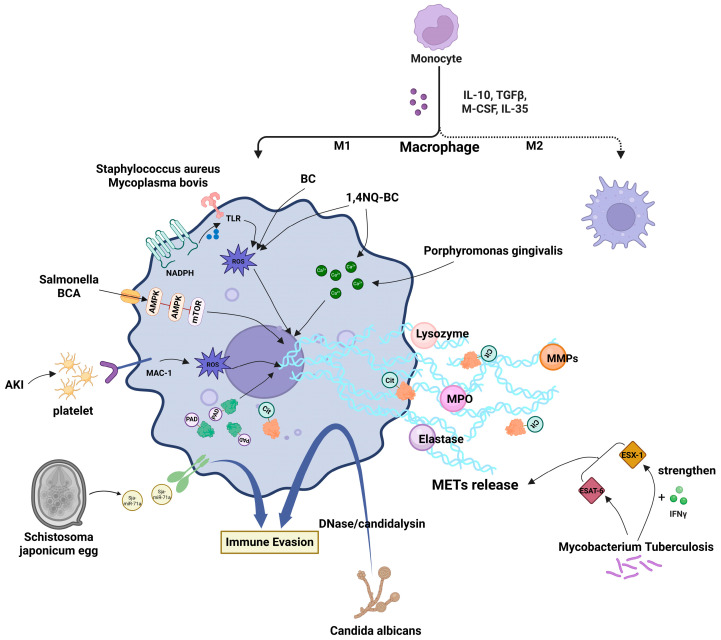
Mechanisms of multiple factors inducing macrophage extracellular traps and related targeted drugs.

In addition to promoting necroptosis, charged polyhedral oligomeric silsesquioxane has been shown to induce the formation of METs by activating oxidative stress and autophagy pathways [36]. Meanwhile, the effect of pollutants on macrophage polarization states should be noted. M1-polarized macrophages, upon exposure to pollutants, release significantly higher levels of METs compared to M2-polarized macrophages [17]. This finding highlights the critical role of macrophage polarization in shaping immune responses to environmental pollutants. Environmental pollutants influence macrophage immune function by modulating MET formation through multiple mechanisms, including the induction of oxidative stress, autophagy, and programmed necrosis [17,36,37,38,39]. Future studies should further elucidate these pathways to identify novel strategies for mitigating pollutant-induced immune dysregulation and the progression of chronic inflammatory diseases (Figure 1).

## 4. METs in the Pathogenesis of Various Diseases

### 4.1. Infectious Diseases

Recent studies have demonstrated that *E. coli*, the primary pathogen in bladder infections [40], promotes the formation of METs during infection. Li et al. identified a subset of CX3CR1hi macrophages located perivascularly beneath the bladder urothelium, termed sub-urothelial perivascular macrophages (suPVMs) [6]. In a uropathogenic *E. coli* infection model, genetic knockout and pharmacological depletion of suPVMs facilitated bacterial translocation into the bloodstream and led to bacteremia. This finding highlights the critical role of suPVMs as an essential bladder–blood immune barrier that restricts the hematogenous spread of uropathogens. Furthermore, suPVMs released METs that trap bacteria within the urothelial layer. METs produced by suPVMs also contained MMP13, which promoted the recruitment of neutrophils for bacterial clearance [6]. During recurrent infections, monocyte-derived replenishment of suPVMs helped to restore the bladder’s immune barrier, indicating a dual role of suPVMs in maintaining vascular integrity and limiting pathogen dissemination [6].

### 4.2. Respiratory Diseases

METs have emerged as key contributors to the pathogenesis of chronic inflammatory respiratory diseases, such as asthma and pulmonary fibrosis [21,41]. Several studies have highlighted the pivotal role of METs in airway inflammation among patients with severe asthma (SA) [41,42]. Analysis of clinical samples has revealed that classical monocytes and M1-polarized macrophages secreted high levels of METs in SA patients, leading to the amplification of airway inflammation by activating neutrophils and innate lymphoid cells [41]. Furthermore, more production of METs was associated with increased levels of inflammatory markers, including monocyte chemoattractant protein-1 and soluble suppression of tumorigenicity 2 (sST2), as well as increased peripheral neutrophil counts [41]. Notably, the administration of IL-33/ST2-neutralizing antibodies and PAD inhibitors (e.g., YW3-56) significantly attenuated MET release, suggesting that both the IL-33/ST2 signaling axis and PAD-dependent mechanisms represent promising therapeutic targets in asthma [42].

Kummarapurugu et al. reported that neutrophil elastase (NE) was internalized by macrophages via endocytosis in cystic fibrosis (CF), retaining proteolytic activity and subsequently inducing the release of METs [21]. The underlying mechanism involves NE-mediated citrullination and cleavage of histone H3, which facilitates MET formation and exacerbates airway inflammation in CF patients [21]. In addition, Navarro et al. initially reported the presence of METs in the lung tissue of patients with Hermansky–Pudlak syndrome-associated pulmonary fibrosis (HPS-PF) [43]. This study demonstrated that extracellular traps (ETs) released by macrophages contributed to pulmonary inflammation and fibrosis. These findings provide novel insights into the pathogenesis of HPS-PF and suggest that extracellular trapsosis (ETosis) may represent a potential pathogenic mechanism. Further investigation of this process may facilitate the development of new therapeutic strategies for HPS-PF.

### 4.3. Autoimmune Diseases

In recent years, increasing attention has been paid to the role of METs in autoimmune diseases. Two studies demonstrated that, in the development of rheumatoid arthritis (RA), macrophages enhanced citrullination and promoted the generation of autoantibodies by releasing METs, such as anti-citrullinated peptide antibodies (ACPAs) [14,44]. In both collagen-induced arthritis mouse models and synovial biopsy specimens from RA patients, macrophages were found to mediate citrullination via PAD4 expression within secondary lymphoid organs and ectopic lymphoid structures adjacent to the joints [14]. PAD4 expression and its extracellular release were significantly associated with ACPA production, whereas macrophage depletion resulted in a marked reduction in both citrullination levels and serum ACPA concentrations [14]. Researchers have found that METs contributed to the immunopathogenesis of RA by releasing citrullinated antigens, which activated follicular dendritic cells and B cells, thereby promoting the production of ACPAs and sustaining the autoimmune response in RA [14].

Similarly, Shen et al. demonstrated the critical involvement of PAD4 in the development of type 1 diabetes (T1D) [45]. PAD4 facilitated the formation of METs, which promoted the migration of gut-derived T cells, such as Th1 and Tc1 subsets, toward the pancreas, thereby initiating autoimmune responses that lead to T1D onset [45]. Experimental studies in non-obese diabetic (NOD) mice and PAD4-knockout NOD (PAD4−/−NOD) mice found that PAD4 deficiency resulted in a significant reduction in the number of intestinal M1 macrophages and MET formation, leading to suppressed migration of gut-derived T cells and a delay in T1D progression [45]. Researchers demonstrated that M1-polarized macrophages could release C-X-C Motif Chemokine Ligand 10 (CXCL10),which is able to regulate the recruitment of immune cells, antiviral defense and tumor immunity by promoting the migration of gut-derived inflammatory T cells to pancreatic islets through binding to CXCR3 (the homologous receptor expressed on T cells) [46,47]. During MET formation, PAD4 could epigenetically enhance CXCL10 transcription and modulate T-cell migration [45]. In NOD mice, CXCR3 antagonist (AMG487) showed the ability to transiently delay T1D onset, reduce disease severity, attenuate colonic inflammation, and decrease pancreatic lymph node infiltration by gut-derived T cells [45].

### 4.4. Metabolic Diseases

METs play a significant role in the immune responses associated with type 2 diabetes mellitus (T2DM) and insulin resistance. Zhang et al. demonstrated that the silencing of the hepatic hepcidin gene could alleviate inflammation and insulin resistance in the adipose tissue of db/db mice, primarily through the inhibition of MET formation [48]. Hepcidin knockdown led to a marked reduction in macrophage infiltration and MET generation in adipose tissue, accompanied by improvements in insulin signaling pathways, involving Insulin Receptor Substrate-1, Protein Kinase B, and Glycogen Synthase Kinase-3β.

Pertiwi et al. identified macrophages, neutrophils, mast cells, and eosinophils exhibiting ETs in coronary artery specimens from acute myocardial infarction patients [49]. NETs were predominantly observed in fresh and lytic thrombi, whereas METs were more prominent in organized thrombi [49]. These findings suggest that NETs and METs contribute to the pathogenesis of atherothrombosis by promoting thrombus formation in the early stages and enhancing thrombus stabilization during later stages of organization. However, the mechanisms by which METs contribute to atherosclerotic thrombus development remain insufficiently understood and warrant further investigation.

### 4.5. Tumors

METs have been implicated in promoting malignant progression by modulating the tumor immune microenvironment. In hepatocellular carcinoma (HCC), Huang et al. reported that sorafenib induced MET formation via the Rho GDP dissociation inhibitor gamma (ARHGDIG)/IL-4/PADI4 axis, which led to the inhibition of ferroptosis and the development of sorafenib resistance [50]. Both in vivo and in vitro experiments revealed that sorafenib upregulated the expression of ARHGDIG (Rho GDP dissociation inhibitor gamma), promoted IL-4 secretion by HCC cells, activated PADI4, and induced MET formation by M2-polarized macrophages [50]. METs could suppress ferroptosis, thereby protecting tumor cells from the therapeutic effects of sorafenib and contributing to drug resistance [50]. The study further demonstrated that co-treatment-of sorafenib with DNase I or an IL-4 neutralizing antibodies effectively eliminated METs and improved the therapeutic efficacy of sorafenib, as evidenced by reducing tumor growth and prolonging survival in mouse models [50].

In pancreatic cancer, Liao et al. found that Mixed Lineage Kinase Domain-like Protein (MLKL) mediating necroptosis not only upregulated CD47 expression on tumor cell surfaces to inhibit macrophage-mediated phagocytosis, but also promoted epithelial–mesenchymal transition and endothelial adhesion through the release of CXCL8 and activation of the CXCR1/2 signaling axis [51]. This mechanism markedly enhances the metastatic potential of pancreatic cancer cells, particularly to the liver [51].

A similar mechanism has been observed in colorectal cancer, where METs were highly enriched in tumor tissues. METs promote invasive tumor growth by entrapping cancer cells and degrading extracellular matrix components [52]. Higher MET levels are independently associated with a worse prognosis in CRC patients. MET formation is linked to distant metastasis and inflammatory markers, and its induction enhances colorectal cancer cell invasiveness. Moreover, colorectal cancer cells could further promote MET formation, establishing a positive feedback loop [52]. This interaction could be disrupted by PAD2-IN-1 (a selective PAD2 inhibitor), which impairs the crosstalk between colorectal cancer cells and METs and suppresses liver metastasis, thereby presenting a potential therapeutic strategy [52].

In glioblastoma, a previous study involving 15 specimens characterized macrophage subtypes and examined the presence of METs [53]. The results showed that M1 macrophages predominated in normal brain regions and tumor margins, while M2 macrophages were significantly increased in tumor core regions, accounting for over 85% of infiltrating macrophages [53]. Most of the M2 macrophages were in an activated or phagocytic state. METs were primarily localized at the interface between the tumor core and necrotic zones, where fibrinogen deposition suggested involvement in inflammatory exudation and fibrotic structure formation [53]. However, the specific roles and underlying mechanisms of M2 macrophages and METs in the glioblastoma microenvironment remain to be elucidated.

### 4.6. Transplantation and Tissue Injury

Guo et al. reported that high levels of donor-derived cell-free DNA (ddcfDNA) following kidney transplantation were positively correlated with macrophage infiltration and the formation of METs within the graft [54]. METs exacerbated Banff lesions, such as interstitial fibrosis and tubular atrophy, by releasing pro-inflammatory cytokines, including IL-6 and TNF-α, ultimately impairing graft function [54]. In a previous study on hepatic ischemia-reperfusion injury (IRI), Wu et al. found that patients undergoing hepatic resection and mice subjected to hepatic IRI both exhibited increased MET formation and hepatocyte ferroptosis [5]. Suppression of MET formation and administration of iron chelators significantly alleviated post-ischemic liver injury. Mechanistically, METs could impair the phagocytic function of macrophages, leading to the intracellular accumulation of iron and free fatty acids in hepatocytes, thereby triggering ferroptosis [5]. Okubo et al. explored the role of METs in trauma-induced rhabdomyolysis and the associated pathogenesis of acute kidney injury (AKI) [14]. They demonstrated that platelet activation during rhabdomyolysis promoted MET formation through macrophage antigen-1 (Mac-1) and ROS-dependent pathways [14]. This process was closely associated with the severity of tubular damage. Inhibition of Mac-1 or PAD4 significantly reduced MET formation and mitigated renal injury, highlighting these pathways as potential therapeutic targets [14].

METs also play a pivotal role in the progression of ocular trauma-related diseases. In age-related macular degeneration (AMD), microglia and macrophages are critical for tissue repair and inflammatory regulation. Conedera et al. provided insights into the dynamic coordination of these processes [55]. In a laser-induced retinal injury model in mice, macrophages were observed to aggregate and form ETs in the photoreceptor (PR) layer on days one and seven post-injury. Treatment with CI-amidine and a PAD4 inhibitor, reduced ET formation, accelerated retinal repair, and improved vascular barrier function [55]. ETs also influenced the inflammatory microenvironment. Inhibition of ETosis led to a phenotypic shift in microglia from a pro-inflammatory type to a phagocytic type [55]. Macrophage recruitment exacerbated tissue damage and contributed to the chronic progression of AMD [55]. These findings suggest that ETs act as both amplifiers of inflammation and barriers to tissue regeneration. Targeting ETosis, such as through PAD4 inhibition, may offer a promising strategy to reshape immune responses, suppress chronic inflammation, and promote retinal regeneration.

### 4.7. Others

In 2013, Mohanan et al. identified macrophage infiltration and the formation of crown-like structures (CLS) around dead adipocytes in adipose tissue from obese patients [56]. This phenomenon was associated with elevated levels of inflammatory cytokines such as TNF-α and IL-1β. The study proposed that macrophages within CLS might undergo PAD2-mediated citrullination of histone H4 at arginine 3 (H4R3), leading to the formation of METs and exacerbation of local inflammation [56]. After seven years, Zambrano et al. provided the evidence of ETs in the semen of patients with acute epididymitis, revealing a strong correlation between ETs levels and infection severity [57]. The authors suggested that ETs might impair sperm function by releasing toxic enzymes such as elastase and might act synergistically with reduced fructose levels to further deteriorate semen quality [57]; However, the underlying mechanisms remain to be fully elucidated.

**Figure 2:**
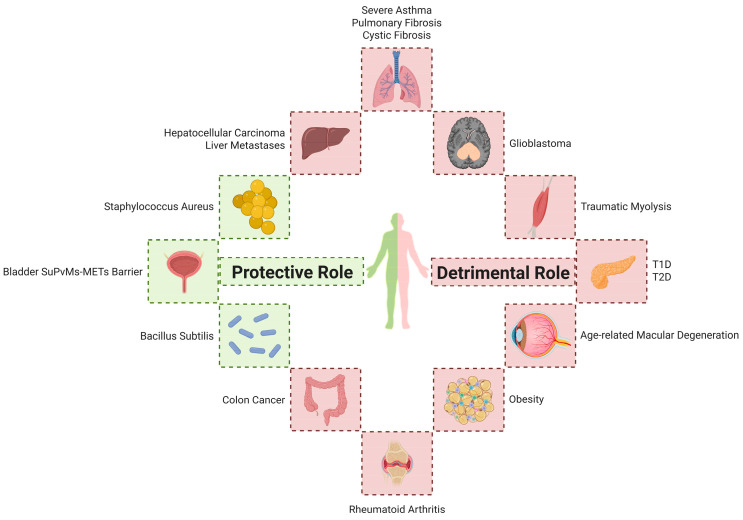
METs play a double-edged role in multiple diseases.

These findings underscore the significant role of METs in various disease contexts. Future research should aim to elucidate the regulatory mechanisms governing MET formation and explore therapeutic strategies targeting METs, which may offer novel insights into the treatment of MET-associated pathologies (Figure 2).

## 5. Therapeutic Strategies Targeting Macrophage Extracellular Trap Mechanisms

Recently, preliminary studies have explored therapeutic agents targeting METs in the context of various diseases. Many studies have investigated the effects of different compounds on MET formation and their roles in modulating immune responses [13,58,59]. Evidence suggests that modulating the generation or function of METs holds the potential to enhance host immune defense, suppress excessive inflammatory responses, and mitigate tissue damage. As mentioned previously, BCA could promote autophagy through the AMPK/ULK1/mTOR signaling pathway, enhancing macrophage-mediated defense against *Salmonella’*s infection [13]. Additionally, BCA improved immune responses by reversing Spleen Focus Forming Virus Proviral Integration Oncogene 1 (SPI1) dependent M2 polarization of macrophages [13].

In 2023, cannabidiol (CBD) was reported to reduce hepatic MET formation induced by perfluoroctane sulfonate (PFOS) via modulation of the PAD4/Coiled-Coil Domain Containing Protein 25 (CCDC25)/Integrin-Linked Kinase (ILK)/NF-κB signaling pathway, thereby improving immune function [60]. Shen et al. demonstrated that fosfomycin (FOM) promoted ETs formation in both neutrophils and macrophages and increased ROS accumulation, leading to improved bactericidal activity against *S. aureus* [59]. Chow et al. found that statins suppressed macrophage phagocytic activity while significantly promoting MET formation, which enhanced extracellular antimicrobial activity and facilitated the clearance of *S. aureus* [1]. In conclusion, these findings underscore the promise of MET-targeted therapeutics in the treatment of infectious and inflammatory diseases. Modulating the formation or function of METs may enhance host immunity, mitigate pathological inflammation, and reduce tissue injury.

Nanodrug targeting strategies show promising progress. Tongyu Zhang et al. engineered DNase I-functionalized nanoparticles for cerebrovascular NETs [61]. This system conferred endothelial protection and reduced immunothrombosis by degrading intravascular NETs components. Furthermore, it attenuated neuronal damage through ROS scavenging within cerebral vasculature, decreased infarct volume, and enhanced blood-brain barrier integrity [61]. Yuwei Zhao et al. developed macrophage membrane-coated polydopamine nanoparticles (MM@mPDA-PM NPs) that significantly suppressed NETs formation by downregulating myeloperoxidase (MPO), NE, and PAD4 production [58]. These nanoparticles could also promote M2 macrophage polarization through the inhibition of nuclear factor kappa-light-chain-enhancer of activated B cells (NF-κB) and Janus kinase-signal transducer and activator of transcription (JAK-STAT) pathway, thus modulating inflammatory cascades and showing therapeutic potential for acute lung injury (ALI) [58]. Zhiwei Zhang et al. constructed glycoRNA-enriched neutrophil membrane vesicles encapsulating small interfering RNA targeting metallothionein 1 (siMT1), which attenuated abdominal aortic aneurysm progression by disrupting NETs-mediated inflammatory cascades [62]. Although no nanotherapeutic approaches currently exist for METs modulation, nanotechnology represents a promising frontier for developing novel METs-directed therapeutics (Table 1).

## 6. Conclusions

Since the first report in 2010, METs have emerged as a significant focus in immunological research. Substantial advances have been achieved in understanding the mechanisms of METs formation, the functional roles in diverse pathological conditions, and the promise as therapeutic targets; however, numerous challenges remain unresolved. Future studies should aim to further dissect the regulatory pathways underlying MET formation, with particular emphasis on roles in diverse pathological conditions. Moreover, the development of MET-targeted therapies must carefully account for the dualistic nature to achieve therapeutic precision and minimize adverse effects. As our understanding of the functions and regulatory mechanisms of METs deepens, MET-targeted strategies are expected to offer novel therapeutic breakthroughs as well as improve clinical outcomes and quality of life for patients.

## Figures and Tables

**Table 1:** Drugs targeting extracellular traps.

Action Direction	Agents/Strategies	Key Mechanism	Target/Indication
Inhibit METs Formation/Release	PAD inhibitors (CI-amidine, YW3-56, PAD2-IN-1)	Block histone citrullination	RA, SA, T1D [14,42,44,45]
	Neutralizing antibodies (anti-IL-4/anti-IL-33/anti-ST2)	Inhibit IL-33/ST2 signaling	Asthma [42]
	Mac-1 inhibitors	Block platelet-macrophage interaction	Rhabdomyolysis-induced AKI [14]
	Iron chelators	Reduce iron overload	Hepatic IRI [5]
	Cannabidiol (CBD)	Modulate PAD4/CCDC25/ILK/NF-κB pathway	Reduce PFOS-induced hepatic METs [60]
Enhance Defensive METs	Biochanin A (BCA)	Activate AMPK/ULK1/mTOR autophagy; reverse SPI1-dependent M2 polarization	Anti-*Salmonella* defense [13]
	Statins	Suppress phagocytosis; promote MET release	Clearance of *S. aureus* [1]
Enhance ETs Bactericidal Activity	Fosfomycin (FOM)	Promote ETs formation; increase ROS accumulation	Anti-*S. aureus* activity [59]
Indirect Modulation	CXCR3 antagonist (AMG487)	Block CXCL10/CXCR3 axis	Delay T1D onset (transient effect) [45]
	Hepcidin gene silencing	Reduce macrophage infiltration	Improve insulin resistance in T2DM [48]
NETs-Targeted Nanotherapies	DNase I-functionalized NPs	Degrade NETs-DNA; scavenge ROS	Cerebrovascular protection [50]
	Macrophage membrane-coated polydopamine NPs (MM@mPDA-PM NPs)	Downregulate MPO/NE/PAD4; promote M2 polarization	ALI [58]
	GlycoRNA-enriched neutrophil membrane vesicles	Deliver siMT1 to disrupt NETs-mediated inflammation	Abdominal aortic aneurysm [62]
Research Gap	MET-targeted nanotherapies	No existing strategies	Future development frontier

## Data Availability

Not applicable.

## Abbreviations

The following abbreviations are used in this manuscript:

Abbreviation	Full Name	Abbreviation	Full Name
ACPAs	Anti-Citrullinated Peptide Antibodies	ESX-1	ESAT-6 Secretion System 1
AKI	Acute Kidney Injury	ETs	Extracellular Traps
ALI	Acute Lung Injury	HCC	Hepatocellular Carcinoma
ARHGDIG	Rho GDP Dissociation Inhibitor Gamma	IFN-γ	Interferon-gamma
BC	Black Carbon	IL-4/6/10/33	Interleukin-4/6/10/33
BCA	Biochanin A	ILK	Integrin-Linked Kinase
BMDMs	Bone Marrow-Derived Macrophages	IP-10	Interferon γ-induced Protein 10
CCDC25	Coiled-Coil Domain Containing Protein 25	IRI	Ischemia-Reperfusion Injury
CF	Cystic Fibrosis	IRS-1	Insulin Receptor Substrate-1
citH3	Citrullinated Histone H3	LPS	Lipopolysaccharide
CLS	Crown-Like Structures	METs	Macrophage Extracellular Traps
CXCL10	C-X-C Motif Chemokine Ligand 10	METosis	Macrophage Extracellular Traps-associated Cell Death
CXCR3	C-X-C Chemokine Receptor Type 3	MMPs	Matrix Metalloproteinases
ddcfDNA	Donor-Derived Cell-Free DNA	mTOR	Mechanistic Target of Rapamycin
E-EVs	Egg-derived Extracellular Vesicles	NADPH	Nicotinamide Adenine Dinucleotide Phosphate
ELs	Ectopic Lymphoid Structures	NE	Neutrophil Elastase
ESAT-6	6kDa Early Secretory Antigen Target	NETs	Neutrophil Extracellular Traps
PADs	Peptidyl Arginine Deiminases	NOD	Non-Obese Diabetic
PFOS	Perfluorooctane Sulfonate	NTHi	Nontypeable *Haemophilus influenzae*
PMA	Phorbol Myristate Acetate	SA	Severe Asthma
PPAR-γ	Peroxisome Proliferator-Activated Receptor gamma	SPI1	Spleen Focus Forming Virus Proviral Integration Oncogene 1
PR	Photoreceptor	sST2	Soluble Suppression of Tumorigenicity 2
ROS	Reactive Oxygen Species	suPVMs	Sub-urothelial Perivascular Macrophages
HPS-PF	Hermansky–Pudlak Syndrome-associated Pulmonary Fibrosis	T1/2D	Type 1/2 Diabetes
TNF-α	Tumor Necrosis Factor-alpha		
